# Musicality and Age Interaction in Tone Development

**DOI:** 10.3389/fnins.2022.804042

**Published:** 2022-02-21

**Authors:** Nari Rhee, Aoju Chen, Jianjing Kuang

**Affiliations:** ^1^Department of Linguistics, University of Pennsylvania, Philadelphia, PA, United States; ^2^Utrecht Institute of Linguistics OTS, Utrecht University, Utrecht, Netherlands

**Keywords:** musicality, tone, pitch, production, development, cue integration

## Abstract

Vocal pitch, which involves not only F0 but also multiple covarying acoustic cues is central to linguistic perception and production at various levels of prosodic structure. Recent studies on language development have shown that differences in learners' musicality affect the F0 cue development in perception of sentence-level intonation or in prosodic realization of focus. This study aims to contribute toward a fuller understanding of the effect of musicality on linguistic pitch development via a close investigation of the relationship between musicality, age, and lexical tone production covering both F0 and spectral cues in children. Forty-three native Mandarin-speaking children between the ages of 4 and 6 years are recruited to participate in both a semi-spontaneous tone production task and a musicality test. For each age (4, 5, and 6 years) and musicality (below or above the median score of each age group) group, the contrastivity of the four tones is evaluated by performing automatic tone classification using three sets of acoustic cues (F0, spectral cues, and both). It has been found that higher musicality is associated with higher contrastivity of the tones produced at the age of 4 and 5 years, but not at the age of 6 years. These results suggest that musicality promotes earlier development of tone production only in earlier stages of prosodic development; by the age of 6 years, the musicality advantage in tone production subsides.

## 1. Introduction

Fundamental frequency (F0) or pitch (hereafter pitch) is used at multiple levels of linguistic structure, such as lexical tones, focus marking, and prosodic phrasing. The ability to process and produce pitch cues accurately is thus central to speech perception and production. However, it has long been known that people vary in their aptitudes for pitch-processing, depending on their so-called musicality (i.e., individual's cognitive capability for music, shaped by both innate aptitude and musical training). At the lower end of the musicality spectrum lie individuals with congenital amusia (Peretz, 2001), a neurogenetic disorder that affects the processing of music and is found in 4% of the general population (Kalmus and Fry, 1980). At the other end are those with absolute pitch, the rare ability to label musical notes of a given sound without external reference (Levitin and Rogers, 2005). Most people, however, fall in neither the most advantaged nor the disadvantaged portions of the musicality spectrum; individuals' musical processing abilities improve with age during childhood (Welch, 1998) and can be further developed through musical training and exposure (Besson et al., 2007). Among various musical cues associated with musicality, the pitch-processing aspect of musicality has received much attention from language researchers (e.g., Ayotte et al., 2002; Patel et al., 2008; Yang et al., 2014; Ong et al., 2020), who have been interested in how the general pitch-processing skills transfer to the perception and production of pitch cues used in languages.

### 1.1. Musicality Effect on Linguistic Pitch Perception

Despite the impairments in pitch-processing, low musicality does not completely compromise linguistic pitch processing during speech perception. An earlier study (Ayotte et al., 2002) has shown that native Quebec-French and English speakers with musical deficits (low music discrimination and recognition) are still able to process speech intonation. However, multiple studies have subsequently reported that with careful manipulation of the stimuli, significant differences are observed between those with and without amusia in their ability to perceive pitch cues used in their native languages. For example, German (Hamann et al., 2012), Canadian French (Patel et al., 2008; Hutchins and Peretz, 2012), and English (Patel et al., 2008; Liu et al., 2010) listeners with amusia are worse than non-amusics at discriminating statements from questions on the basis of the sentence-final intonational contours. Similarly, musicians outperform non-musicians in detecting weak F0 manipulations in both music and speech (French), with better discrimination accuracy and shorter onset latency in the brain electrical activity (event-related potentials) (Schön et al., 2004). Musicality also influences lexical tone perception, which involves sensitivity to more local pitch variations. Native speakers of Mandarin (Nan et al., 2010; Yang et al., 2014) and Cantonese (Liu et al., 2016; Ong et al., 2020) with lower musicality are less accurate than the controls in tonal discrimination, and show reduced categorical perception of tones (Mandarin: Jiang et al., 2012; Cantonese: Zhang et al., 2017). The mild yet significant effect of musicality on the perception of pitch cues is thus observed cross-linguistically at both intonational and tonal levels.

### 1.2. Musicality Effect on Linguistic Pitch Production

While impairments in pitch perception often lead to less proficient singing and worse performance on non-linguistic pitch-matching tasks, the effect of musicality on native speakers' production of linguistic pitch cues is minimal, if any. Sentence intonation imitation studies report that speakers with or without amusia can imitate the heard intonational pitch contours in their native languages with little (for British English, Liu et al., 2010) to no (for Quebec French, Hutchins and Peretz, 2012) differences in accuracy. Similarly, speakers of tonal languages with lower musicality produce lexical tones with F0 contour, F0 excursion size, and tonal intelligibility comparable to the controls' (Mandarin: Nan et al., 2010; Yang et al., 2014; Cantonese: Liu et al., 2016; Ong et al., 2020). Consequently, questions arise regarding why speakers with low musicality have more intact linguistic pitch production than perception. The source of this asymmetry in the effect of musicality on native language pitch perception and production has been hotly debated (e.g., Loui et al., 2008, 2009; Nan et al., 2010; Hutchins and Peretz, 2012), but no consensus has yet been reached.

### 1.3. Musicality and Language Development

One important step toward fully understanding the influence of musicality on linguistic pitch cues is to explore how musicality affects the process of language learning. The prevalent idea that musicality facilitates language learning has been tested by many language acquisition researchers. A positive effect of musicality has been reported on various aspects of first language acquisition, including verbal memory (Ho et al., 2003), verbal intelligence, reading (Moreno et al., 2009), vocabulary (Linnavalli et al., 2018), syntactic processing (Jentschke and Koelsch, 2009; Przybylski et al., 2013), phonological awareness (Vidal et al., 2020), and phonological processing (Chobert et al., 2011).

Regarding the development of linguistic pitch cues in a first language, although only a limited number of studies have been conducted, they have generally found that learners with higher musicality indeed have some advantage. For example, musically-trained and untrained 7- to 9-year-olds differ in their ability to detect small discrepancies in the native sentence-final intonation (French: Magne et al., 2006; Portuguese: Moreno et al., 2009). A more recent study on Dutch-speaking 4- to 10-year-olds' prosodic production shows that higher musicality is positively correlated with a more adult-like prosodic realization of focus (i.e., new information in a sentence), though this correlation becomes weaker at older ages (Chen, 2016). Together, these findings suggest a facilitating effect of musicality on the use of prosodic pitch cues over the course of language development.

### 1.4. Pitch Cue Developmental Trajectory

The acquisition of cues for phonological contrasts begins early in the first few years of infancy, but an adult-like mastery of the contrasts is not reached until much later in development. The developmental trajectory of linguistic pitch cues, too, spans over the entire childhood and even into early adolescence (see Prieto and Esteve-Gibert, 2018; Chen et al., 2020 for a review). In the case of lexical tone acquisition, early sensitivity to tonal cues develops as early as 4 months (Yeung et al., 2013), and by 3 years, children produce tones with some F0 contrast (Hua and Dodd, 2000). However, the full mastery of the F0 cues for tones is not achieved until much later in development. Specifically, Mandarin-speaking 3-year-olds do not have an adult-like tone identification (Wong et al., 2005), and children's categorical perception of tones continues to sharpen throughout the age of 4 and 7 years (Chen et al., 2017). A recent study on Mandarin-speaking 4–5, 7–8, and 10–11 year-olds' tonal production has revealed that an adult-like tonal contrast in F0 cues is not produced until the age of 7–8 years (Rhee et al., 2021). While this study has studied a relatively wide age range, it has not examined the tone production of children at the age of 6 years, a critical time in the development of prosody (Ballard et al., 2012; Chen et al., 2017; Wong and Strange, 2017) and pitch (Schneider et al., 1986).

### 1.5. The Current Study

The above-reviewed literature has suggested that for the adults, musicality has an asymmetric effect on linguistic pitch perception and production, whereas for children, higher musicality leads to earlier pitch cue development in both production (for focus-marking, Chen, 2016) and perception (for intonation, Magne et al., 2006; Moreno et al., 2009). However, often missing in the discussion of pitch development is the fact that the production and perception of vocal pitch involves more than just the F0 height and contour. In fact, the perception of pitch involves a highly multidimensional set of covarying cues, including F0, spectral tilt and noise, duration, and amplitude (see de Cheveigné, 2010 for a review). Among the covarying cues of pitch, spectral cues, which acoustically correlate with voice quality, are known to be particularly integrative and systematic in both pitch perception (Kuang and Liberman, 2018) and production (Titze, 1994; Kuang, 2017), and thus linguistically meaningful as an enhancement cue (e.g., Mandarin: Belotel-Grenié and Grenié, 1994; Cantonese: Yu and Lam, 2014; Northern Vietnamese: Brunelle, 2003; English and Spanish: Garellek, 2014, 2015). Linguistic pitch development spans over the entire childhood and exhibits different developmental trajectories for different covarying psychoacoustic cues of pitch (i.e., F0 or spectral cues) and their integration (i.e., the combination of both F0 and spectral cues). Specifically, Rhee et al. (2021) has found that Mandarin-speaking children's spectral cues continue to develop even after the full mastery of the primary F0 cues (by the age of 7–8 years) and do not reach an adult-like tonal contrastivity even at the age of 10–11 years. Likewise, the tonal contrastivity with both F0 and spectral cues increases throughout childhood, but not to the level of adults. Yet no study has examined how cognitive factors such as musicality influence the development of each covarying cue of pitch.

The current study aims to contribute toward a fuller understanding of the effect of musicality on linguistic pitch development via a close investigation of the relationship between musicality, age, and lexical tone production covering both F0 and spectral cues in children. To this end, we analyzed the musicality development by age, and examined how age and musicality influence the usefulness of various pitch cues (i.e., F0, spectral cues, or both) in manifesting the contrastivity of lexical tones, through computational modeling of children's tone production via automatic tone classification. For each age and musicality group, supervised machine-learning classification algorithms assessed how successful each set of pitch cues was at classifying the tonal categories. Our analysis focused on Mandarin-speaking children aged 4–6 years, an important period for prosodic development. For a more direct comparison with older children in later stages of language development, we have adopted 7- to 8-year-olds' tone production data from Rhee et al. (2021).

## 2. Methods

### 2.1. Procedures

#### 2.1.1. Participants

A total of 43 typically-developing monolingual Mandarin-speaking children between the age of 4–6 years (range: 4;0–6;11) were recruited from Beijing twenty-first century Kindergarten to participate in a speaking (tonal production) and a listening (musicality testing) session. The sessions took place on different days; each child always did the speaking session before the musicality session.

#### 2.1.2. Tone Production

Following Yang and Chen (2018), 80–160 SVO sentences were semi-spontaneously elicited from each speaker, using a picture-matching game (Chen, 2011). The participants were asked to respond to the experimenter's question about a given picture (e.g., the subject, the verb, the object, or the whole scene) by describing the picture or by correcting the experimenter's descriptions, in full SVO sentences (e.g., 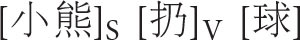
_O_; “The little bear throws the ball”). The target sentences consisted of words varying in the four tones of Mandarin in each sentence position.

Acoustic cues were extracted from 9 time-normalized subsegments of the sentence-medial monosyllabic verbs using VoiceSauce (Shue et al., 2011). F0 was extracted using STRAIGHT (Kawahara et al., 1999). In addition to F0, we extracted information about the covarying voice quality cues through spectral cues. Various aspects of voice quality can be acoustically measured via information in the spectrum (e.g., Holmberg et al., 1995; Blankenship, 1997; Hanson et al., 2001; Esposito, 2010), through measures such as Cepstral Peak Prominence (CPP), a measure of aperiodicity in the signal, and relative amplitude differences of the lower and higher harmonics (H1^*^-H2^*^, H2^*^-H4^*^, H1^*^-A1^*^, H1^*^-A2^*^, H1^*^-A3^*^, H4^*^-2K^*^, 2K^*^-5K^*^, corrected for the influence of formant frequencies and bandwidths on the harmonics; Iseli et al., 2007). To eliminate the effect of different onset consonants, measurements from the first 3 subsegments were removed from analysis.

All extracted measures were min-max normalized by speaker and recording session. Due to the interactive and spontaneous nature of the task, tokens with loud background noises, wrong target words, or octave jumps in F0 tracking were systematically and manually identified and removed.

Additionally, to illustrate how the 4- to 6-year-old children's production data compared to older children's, we adopted production data from 10 7- to 8-year-olds from Rhee et al. (2021), collected using the same design but without musicality testing.

The number of speakers and tokens from all participants are summarized in Table 1^1^.

**Table 1:** The number of speakers and tokens in each age group.

**Age (years)**	**No. of speakers**	**No. of tokens**
4	10	296
5	13	390
6	20	1,061
7–8^*^	10	589

* *Production data from children of 7–8 years of age were adopted from Rhee et al. (2021) for comparison*.

#### 2.1.3. Musicality Testing

The participants' musicality was assessed using the tonal subset of the Primary Measures of Music Audiation (PMMA), a test designed to measure the musical aptitude of children in primary grades (Kindergarten to Grade 3) independent of musical training (Gordon, 1979). For each question, participants were instructed to listen to two short melodies and indicate whether the melodies were identical. Two example questions were given prior to the test as training. Given the short attention spans of younger children, 4-year-olds were given 20 questions, which took about 10 min to complete. Older children were given the full test set of 40 questions, taking approximately 20 min.

### 2.2. Analysis

In this section, the musicality and tone production data were analyzed to study the influence of children's musicality on the development of linguistic pitch cues.

#### 2.2.1. Musicality and Age

Each participant's raw musicality test score in percentage was calculated by dividing the number of correct responses by the total number of questions given. To test the effect of age on musicality, the raw test score distributions of the 4-, 5-, and 6-year-olds were compared, using the Kruskal-Wallis and *post-hoc* Dunn's tests for non-parametric comparisons of non-normally distributed data. Furthermore, to establish whether the participants had typical musicality for their ages, the percentile rank norms provided in the manual (Gordon, 1979) from typically-developing English-speaking children in the United States were used to convert the test scores into grade-based percentile ranks of the age groups (Kindergartener for 4- and 5-year-old participants, and Grade 1 for the 6-year-olds).

#### 2.2.2. Tone Contrastivity

The effect of musicality on the development of linguistic pitch production was tested by investigating whether children's musicality influenced their ability to produce distinctive pitch cues that manifest the tonal contrast. In particular, three sets of pitch cues were assessed for tonal contrastivity: the primary F0 cue, covarying spectral cues (CPP, H1^*^-H2^*^, H2^*^-H4^*^, H1^*^-A1^*^, H1^*^-A2^*^, H1^*^-A3^*^, H4^*^-2K^*^, 2K^*^-5K^*^), and the integration of both F0 and spectral cues. To computationally model how the highly multidimensional and correlated sets of acoustic cues of pitch manifest the contrastivity of the tones in children's tone production, we employed automatic classification on tones, using the acoustic cues as classification features. Automatic classification was specifically chosen for its usefulness in assessing how well a set of cues (features) could be used to identify class categories.

The reliability of the classification accuracy scores achieved was validated via 100 trials of 10-fold cross-validation. That is, supervised machine-learning classifiers were trained on 90% of the data with a chosen set of features (acoustic cues) and class labels (tones), and tested on the remaining subset (10%). These training and testing trials were repeated 100 times, with the data randomly shuffled in each trial. The final accuracy score was calculated by averaging over the accuracy from 100 trials of classification.

To test for the effect of musicality in each age group, classification was performed with the tone production data of each age group divided into high and low musicality groups, based on each participant's performance on the musicality test. Production data from speakers whose scores were below the median of their age groups were categorized as low musicality (i.e., 4-, 5-, and 6-L), and data from those with scores median or above was categorized as high musicality (i.e., 4-, 5-, and 6-H). Because musicality test data was unavailable for the 7- to 8-year-olds (adopted from Rhee et al., 2021), no split has been made for this group. The median score was used for the split to ensure that despite some skewness in the musicality score distributions (6-year-olds: Shapiro-Wilk statistic *W* = 0.90, *p* = 0.04; skewness = –0.78), the participants of the high and low musicality groups fall in the high and low 50th percentiles, respectively.

Finally, to verify the results across various assumptions about the linearity and organization of the multidimensional data space, three different classification algorithms were used: Linear Discriminant Analysis (LDA, *MASS* package, Venables and Ripley, 2002), Support Vector Machine with radial basis kernel (SVM, *e1071* package, Meyer et al., 2018), and Random Forest (RF, *randomforest* package, Liaw and Wiener, 2002). To test for the age effect on tone contrastivity, we compared the average tone classification accuracy (averaged across musicality subgroups) across the age groups. To test the musicality effect, the average classification accuracy was compared between the high and low musicality subgroups of each age group^2^.

## 3. Results

### 3.1. Musicality and Age

**Figure 1:**
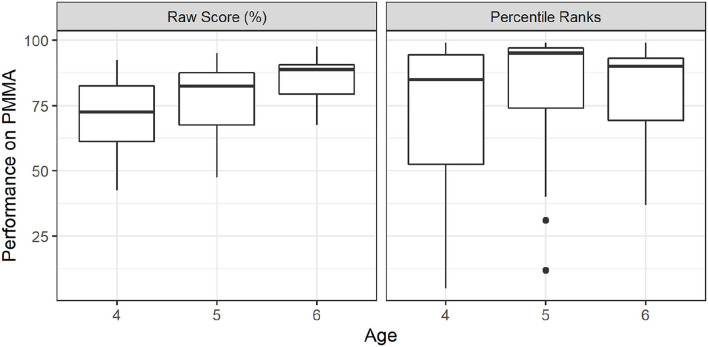
Performance on PMMA musicality test by age. Raw test scores in percentages are shown in the left panel, and grade-based percentile norm ranks in the right. Kruskal-Wallis test on the raw musicality scores yielded significant variation among different ages, [χ^2^_(2,*N* = 44)_ = 6.61, *p* < 0.05]. A *post-hoc* Dunn's test with Holm's multiple comparisons adjustment showed that scores of the 4-year-olds and 6-year-olds differed significantly (*p* < 0.05), but score differences between consecutive age groups were not significant. No significant group differences among the age groups were observed in the percentile norm ranks.

The raw PMMA musicality score and the grade-based percentile rank distributions for each age group are illustrated in Figure 1. The raw test scores (left panel of Figure 1) increased with age, reflecting a general improvement in musicality with age. The raw scores had a median score of 72.50% (mean *M* = 70.68%, *SD* = 16.85) for the 4-year-olds, 82.50% (*M* = 76.35%, *SD* = 15.16) for the 5-year-olds, and 88.75% (*M* = 85.25, *SD* = 8.81) for the 6-year-olds.

A Kruskal-Wallis test on the raw musicality scores yielded significant variation among different ages [χ^2^_(2,*N* = 44)_ = 6.61, *p* < 0.05]. The effect size was moderate (η^2^ = 0.11). A *post-hoc* Dunn's test with Holm's adjustment for multiple comparisons showed that scores of the 4-year-olds and 6-year-olds differed significantly (*p* < 0.05), but score of consecutive age groups were not significantly different.

The grade-based percentile ranks (right panel of Figure 1) resulted in a median percentile rank of 85 (*M* = 49, *SD* = 35.43) for the 4-year-olds, 95 (*M* = 77.92, *SD* = 30.01) for the 5-year-olds, and 90 (*M* = 80.25, *SD* = 19.93) for the 6-year-olds. As all three groups had median percentile ranks well above the 50th percentile, this can be taken as evidence for relatively higher musicality of the participants compared to the typically-developing children population tested in Gordon (1979). However, across the recruited participants, the grade-normalized musicality of each age group was not significantly different [Kruskal-Wallis: χ^2^_(2,*N* = 44)_ = 1.045, *p* = 0.06; a small effect size η^2^ = −0.02], which suggested that although the participants had higher-than-typical musicality, their rate of musicality development with age was typical.

### 3.2. Tonal Classification Accuracy

**Figure 2:**
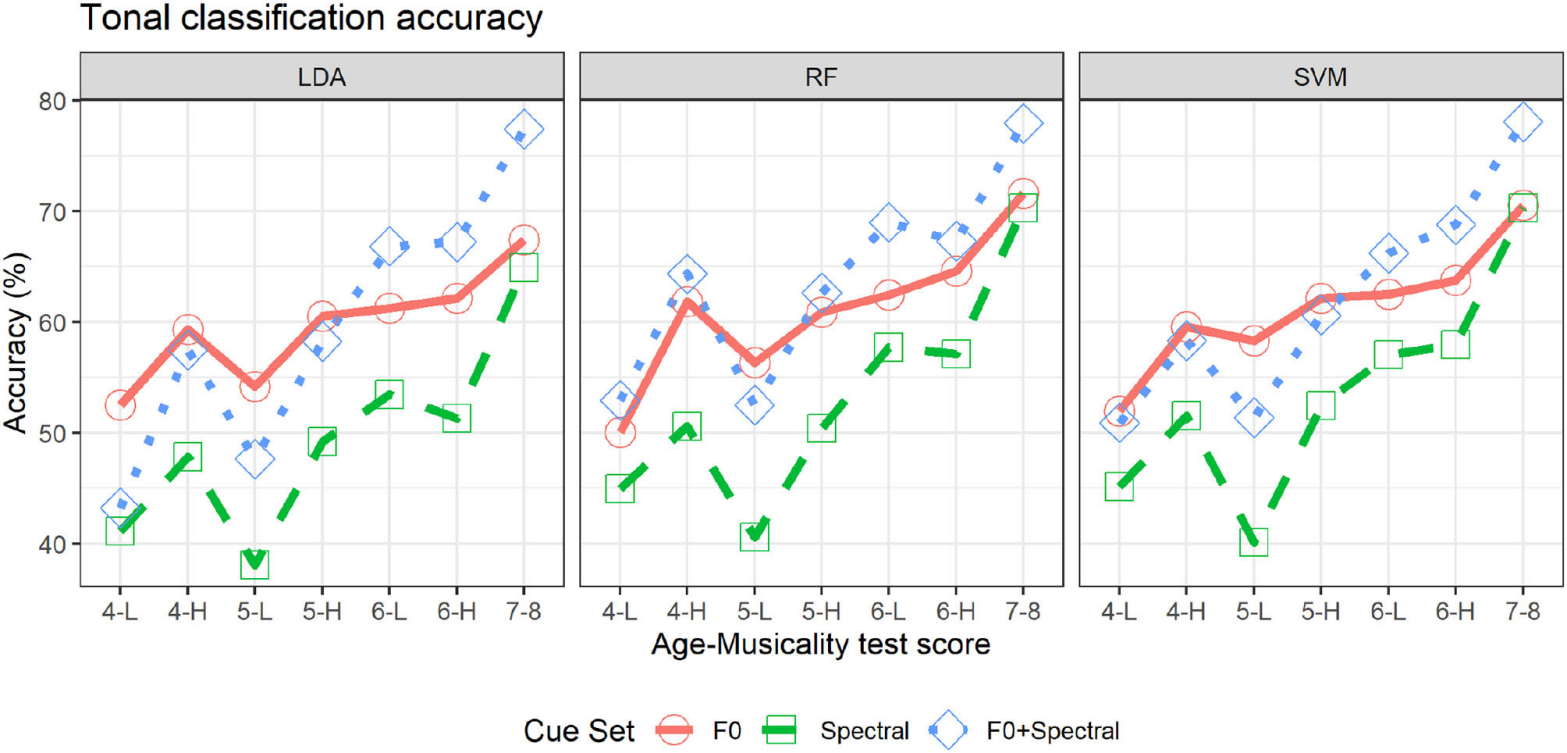
Average tonal classification accuracy from 100 trials of 10-fold cross-validation for each age (4, 5, 6, and 7–8 years) and musicality group (H if median and above, and L if below the median; only available for children between 4 and 6 years of age), using only F0 cues (red solid line), only spectral cues (green dashed line), or both F0 and spectral cues (blue dotted line). Three classification algorithms were tested (LDA: left panel, RF: middle panel, and SVM: right panel).

Accuracy scores of tonal classification using the three supervised machine-learning classification algorithms (LDA, RF, and SVM) are summarized in Figure 2. Results were consistent across classification algorithms, confirming that the overall patterns in the data were robust.

#### 3.2.1. Accuracy Differences by Age

Using F0 cues, the tone classification accuracy gradually increased with age, as shown by the red solid lines for each of the classification algorithms in Figure 2. Averaging across musicality groups, classification using F0 cues achieved an average accuracy of 56% for the 4-year-olds, 57–60% for the 5-year-olds, and 62–64% for the 6-year-olds, depending on the classification algorithm used. The 7- to 8-year-olds had an average accuracy of 67–72%, which was the highest among the tested age groups.

The tone classification accuracy using spectral cues was gradually increasing throughout the tested age groups, except between 4- and 5-year-olds (green dashed lines in Figure 2). The three classification algorithms achieved an average of 45–48% accuracy for the 4-year-olds, 44–46% for the 5-year-olds, 52–57% for the 6-year-olds, and 65–70% for the 7- to 8-year-olds, depending on the algorithm.

Similarly, using both F0 and spectral cues (blue dotted lines in Figure 2), the age-driven increasing trend began from 5-year-olds. The 4-year-olds had an accuracy of 50–59% and 5-year-olds had 53–58%, but 6-year-olds had an average accuracy of 67–68%, and 7- to 8-year-olds had 77–78%.

In addition, comparing the accuracy between cue sets within each age group, using only spectral cues obtained the lowest accuracy for all age groups. Using F0 cues and using both F0 and spectral cues achieved comparable accuracy for the 4- and 5-year-olds, but from the age of 6 years, using both F0 and spectral cues (Age 6: 67–68%, Age 7–8: 77–78%) exceeded the accuracy from using just F0 cues (Age 6: 62–63%, Age 7–8: 69–72%), which revealed that spectral cues provided additive tone information beyond the F0 cues from the age of 6 years.

#### 3.2.2. Accuracy Differences by Musicality

Generally, musicality group differences in tone classification accuracy were larger in younger children than in older children. Classification accuracy using only F0 cues showed the largest musicality group differences in the 4-year-olds. Depending on the algorithm, the 4-H group had classification accuracy 7–12% higher than the 4-L group. In contrast, the 5-year-olds had a smaller improvement with musicality, with the 5-H group achieving accuracy 4–6% higher than the 5-L group. The 6-year-olds exhibited an even smaller effect of musicality: 6-H group achieved accuracy only 1–2% higher than the 6-L group.

Tonal classification accuracy using only spectral cues or using both F0 and spectral cues exhibited a similar pattern in the musicality group differences. Using spectral cues, accuracy differences between musicality groups ranged between 6 and 7% for the 4-year-olds, 10–12% for the 5-year-olds, and 0% for the 6-year-olds. Using both F0 and spectral cues, accuracy differences between musicality groups ranged between 7 and 14% for the 4-year-olds, 9–11% for the 5-year-olds, and –2 to 2% for the 6-year-olds. Except for spectral-cue classification on 5-year-olds' production, musicality-group accuracy differences in tone classification generally decreased with age regardless of the cue set, and by the age of 6 years, the differences were minimal.

Thus, unlike the effect of age, which promoted the higher accuracy of using both F0 and spectral cues over using just F0 cues from the age of 6 years, no noticeable effect of musicality was observed.

## 4. Discussion

This study has presented new data and analysis on the linguistic pitch cue development with a focus on the effect of musicality on the development of various covarying cues of pitch. Using lexical tone production and musicality data from Mandarin-speaking 4- to 6-year-olds, the contrastivity of the tones with different sets of pitch cues (the primary F0 cues, the covarying spectral cues, and the integration of both F0 and spectral cues) has been compared across age (4, 5, and 6 years) and musicality (high or low) groups. In order to juxtapose the current study's findings along the pitch developmental trajectory in lexical tone production, the 7- to 8-year-olds' tone production data from Rhee et al. (2021) has been adopted and reanalyzed.

Prior to analyzing the tone production data, the musicality distribution of the participants at each age was examined. There was a gradual age-driven increase, comparable to the musicality development in typically-developing English-speaking children in Gordon (1979). Since musicality distributions changed with age, the median musicality score for each age group was used to divide the participants into high and low musicality groups.

Putting the current study's results in the light of previous findings of the developmental trajectory of the different pitch cues, we observe that the developmental patterns differ between the primary F0 cue production and the production of covarying spectral cues or its integration. For F0 cues, gradual development is observed in the tested age range of the current study. The tonal contrastivity of F0 cues, which begins to develop in 3-year-olds (Hua and Dodd, 2000), continues to develop between the age of 4 and 6 years and even into 7–8 years, when children finally reach adult-like tonal contrastivity (Rhee et al., 2021). In contrast, the tonal contrastivity using either just spectral cues or both F0 and spectral cues does not increase between the age of 4 and 5 years, but increases between 5, 6, and 7–8 years though not to the level of adults even at the age of 10–11 years (Rhee et al., 2021). Moreover, the combination of F0 and spectral cues do not reach higher tonal contrastivity than just F0 cues until the age of 6 years, suggesting that the integration of spectral cues to enhance the tonal contrast does not begin until 6 years. Taken together, the covarying spectral cues or the integration of both F0 and spectral cues in pitch production exhibit a more delayed development than the primary F0 cues.

Regarding the effect of musicality on the pitch production development, results suggest that musicality does not promote the development of one pitch cue (such as F0 or spectral cues) over another, or facilitate an earlier development of the integration of both F0 and spectral cues. Instead, depending on the stage of language development, musicality may influence the overall development of all pitch cues (F0, spectral, or both cues). For the 4- and 5-year-olds, higher musicality is associated with higher tonal contrastivity regardless of the cue sets of pitch. In contrast, the 6-year-olds exhibit only a minimal difference between the high and low musicality groups, just like adult tone-language speakers who can accurately produce linguistic pitch cues regardless of their musicality (e.g., Nan et al., 2010; Hutchins and Peretz, 2012; Yang et al., 2014). The lack of musicality advantage in older children also resonates with the findings of the study on Dutch focus-marking (Chen, 2016), which has found that older children have weaker correlation between musicality and accurate prosodic marking of focus. One possible explanation for why the musicality effect is observed only during early childhood (4- and 5-year-olds) is that younger children, who have not fully developed the sensorimotor mappings for precise vocal pitch control, tend to rely more on auditory feedback from the perceived pitch during vocalization (Murray and Stepp, 2020). Hence, musicality, the ability to perceive pitch, may cast a larger influence on younger children's ability to produce the local pitch movements in lexical tones.

Some limitations should be considered in interpreting the results. Firstly, the results are based on a small number of participants, especially in the younger (4 or 5 years) children groups. Furthermore, due to the design of the tone contrastivity analysis, continuous variables such as age and musicality have been binned into categories. Hence, a larger-scale study with more participants of broader age and musicality ranges is necessary. Finally, in this study, children's musicality has only been measured via a perception task, while their linguistic pitch cues have been assessed only in production. Hence, future research is also needed to study both musicality and linguistic pitch development from both production and perception sides.

Despite the limitations, this study has presented findings that are key to understanding the interaction between musicality and the development of various linguistic pitch cues. It has shown that young Mandarin-speaking children (4- to 5-year-olds) with higher musicality exhibit expedited development of lexical tone production, where pitch cues have a lexical function and are specified at the syllable level. Future work should extend the current investigation to other prosodic concepts cross-linguistically, such as lexical pitch accents, phrasal pitch accents and focus marking, where linguistic pitch cues are specified differently in the prosodic structure or have different functions than Mandarin lexical tones.

## Data Availability Statement

The raw data supporting the conclusions of this article will be made available by the authors upon request, without undue reservation.

## Ethics Statement

Ethical review and approval were not required for the study on human participants in accordance with the local legislation and institutional requirements. The studies involving human participants were conducted in accordance with the guidelines of ethics at the Utrecht Institute of Linguistics - OTS. Written informed consent to participate in this study was provided by the participants' legal guardian/next of kin.

## Author Contributions

NR and JK designed the study and analyzed the data that AC collected. NR, JK, and AC contributed to the interpretation of the results and to the writing of the manuscript. All authors contributed to the article and approved the submitted version.

## Funding

Data collection was funded by a VIDI grant awarded to AC by the Dutch Research Council (NWO) (grant number 276-89-001).

## Conflict of Interest

The authors declare that the research was conducted in the absence of any commercial or financial relationships that could be construed as a potential conflict of interest.

## Publisher's Note

All claims expressed in this article are solely those of the authors and do not necessarily represent those of their affiliated organizations, or those of the publisher, the editors and the reviewers. Any product that may be evaluated in this article, or claim that may be made by its manufacturer, is not guaranteed or endorsed by the publisher.

## Footnotes

^1^Considering that different sample sizes can affect the results of the automatic classification analysis, we have tested the analysis with a balanced number of participants (*N* = 5) in each group and have confirmed that the results are unaffected.

^2^Accuracy differences are interpreted without additional statistical significance testing because the validity and generalizability of the results have been verified via the 100 trials of 10-fold cross-validation and across different classifying algorithms.
